# Electric pulses used in electrochemotherapy and electrogene therapy do not significantly change the expression profile of genes involved in the development of cancer in malignant melanoma cells

**DOI:** 10.1186/1471-2407-9-299

**Published:** 2009-08-26

**Authors:** Vid Mlakar, Vesna Todorovic, Maja Cemazar, Damjan Glavac, Gregor Sersa

**Affiliations:** 1 Department of Molecular Genetics, Institute of Pathology, Faculty of Medicine, University of Ljubljana, Korytkova 2, SI-1000 Ljubljana, Slovenia; 2 College of Health Care Izola, University of Primorska, Polje 42, SI-6310 Izola, Slovenia; 3 Department of Experimental Oncology, Institute of Oncology Ljubljana, Zaloska cesta 2, SI-1000 Ljubljana, Slovenia

## Abstract

**Background:**

Electroporation is a versatile method for *in vitro *or *in vivo *delivery of different molecules into cells. However, no study so far has analysed the effects of electric pulses used in electrochemotherapy (ECT pulses) or electric pulses used in electrogene therapy (EGT pulses) on malignant cells. We studied the effect of ECT and EGT pulses on human malignant melanoma cells *in vitro *in order to understand and predict the possible effect of electric pulses on gene expression and their possible effect on cell behaviour.

**Methods:**

We used microarrays with 2698 different oligonucleotides to obtain the expression profile of genes involved in apoptosis and cancer development in a malignant melanoma cell line (SK-MEL28) exposed to ECT pulses and EGT pulses.

**Results:**

Cells exposed to ECT pulses showed a 68.8% average survival rate, while cells exposed to EGT pulses showed a 31.4% average survival rate. Only seven common genes were found differentially expressed in cells 16 h after exposure to ECT and EGT pulses. We found that ECT and EGT pulses induce an HSP70 stress response mechanism, repress histone protein H4, a major protein involved in chromatin assembly, and down-regulate components involved in protein synthesis.

**Conclusion:**

Our results show that electroporation does not significantly change the expression profile of major tumour suppressor genes or oncogenes of the cell cycle. Moreover, electroporation also does not changes the expression of genes involved in the stability of DNA, supporting current evidence that electroporation is a safe method that does not promote tumorigenesis. However, in spite of being considered an isothermal method, it does to some extent induce stress, which resulted in the expression of the environmental stress response mechanism, HSP70.

## Background

Electroporation, as a physical method for the delivery of molecules into cells, was developed in 1982 [1]. However, since then it has been developed not only for *in vitro* use but also for *in vivo* use in a variety of applications [2]. Electroporation is of interest as a gene delivery method because, unlike transduction with viruses, it eliminates the risks and limitations linked to the use of viruses. In addition, in spite of extensive research, efficient and safe chemical vectors have not yet been developed for *in vivo *gene delivery [3]. Using appropriate electrical parameters, destabilization of the membrane is reversible, ensuring a high survival of permeabilized cells and the delivery of non-permeant molecules inside the cell, bypassing the normal internalisation route of these molecules [4].

The advantages of electroporation have recently been used by different groups for a novel approach to introducing chemotherapeutics in a variety of tumours, called electrochemotherapy [5-7]. Electrochemotherapy facilitates chemotherapeutic drug delivery into cells by increasing cell membrane permeability under specific electric pulses [4]. It is an effective local treatment for patients with cutaneous and subcutaneous tumour nodules, on the basis of the synergistic association of locally applied electric pulses and low permeant chemotherapeutics such as bleomycin and cisplatin. Moreover, several clinical trials with the same chemotherapeutics showed a good response of melanoma tumour nodules, as well as of other tumour types [5,6,8-10].

As mentioned earlier, electrochemotherapy is not the only application of electroporation. There are an increasing number of applications in which electroporation might be used. Electroporation is frequently used as a method of *in vitro *transfection of genetic materials into prokaryotic or eukaryotic cells. With the development of electric pulse generators, the method has also been used *in vivo *for naked DNA transfection in various rodent tissues, in order to treat various diseases and for vaccination [11-13]. The first clinical trial has also been reported for the treatment of melanoma nodules in patients with plasmid DNA encoding interleukin-12 [14].

The effect of electroporation on the level of cell genetic response has only been studied in muscle cells [15,16]. However, the effect of ECT and EGT pulses on malignant cells have not yet been analysed. In the present work, therefore, we studied the effect of ECT and EGT pulses on human malignant melanoma cells *in vitro*, in order to understand and predict the possible effect of electric pulses on gene expression and their possible effect on cell behaviour.

## Methods

### Cell line

Human malignant melanoma cells SK-MEL28 (HBT-72; American Type Culture Collection, USA) were grown as a monolayer in minimum essential medium (MEM) with Glutamax (Gibco, Paisley, UK), supplemented with 10% fetal bovine serum (FBS; Gibco) and gentamicin (30 μg/mL) (Gibco). Cells were routinely subcultured twice a week and incubated in an atmosphere with 5% CO_2 _at 37°C.

### Electroporation protocol

Confluent cell cultures were trypsinized, washed in MEM with FBS for trypsin inactivation and once in electroporation buffer (125 mM saccharose; 10 mM K_2_HPO_4_; 2.5 mM KH_2_PO_4_; 2 mM MgCl_2_·6H_2_O) at 4°C. The final cell suspension was prepared in electroporation buffer at 4°C, at a concentration of 22 × 10^6 ^cells/mL. Aliquots of the final cell suspension (3 × 10^6 ^cells) were placed between two parallel electrodes with a 2 mm gap and subjected to eight electric pulses for ECT pulses (electric field intensity 1300 V/cm, pulse duration 100 μs and frequency 1 Hz) or eight electric pulses for EGT pulses (electric field intensity 600 V/cm, pulse duration 5 ms and frequency 1 Hz). Electric pulses were generated by a GT-1 electroporator (Faculty of Electrical Engineering, Ljubljana, Slovenia). One aliquot of cell suspension was not subjected to any electric pulses and served as the control treatment. After electroporation, cells were incubated at room temperature for 5 minutes, diluted in MEM with FBS and then plated in culture flasks for 16 h for microarray assay.

### Cell survival after electroporation

Clonogenic assay was used to determine cell survival after electroporation. After exposure to ECT and EGT pulses, SK-MEL28 were plated at a concentration of 500 cells/dish. After 16 days, colonies were fixed, stained with crystal violet and counted. The plating efficiency and the surviving fraction were calculated. The experiments were performed in triplicate and repeated three times.

### RNA extraction

RNA from cells was isolated using TRI REAGENT™ (Sigma Aldrich, St. Louis, USA) and the PureLink™ Micro-to-Midi Total RNA Purification System (Invitrogen, Carlsbad, USA), according to the manufacturer's instructions. Briefly, 16 hours after electroporation, cells were trypsinized, washed in MEM with FBS for trypsin inactivation and resuspended in PBS. After centrifugation at 1500 × g for 5 min, all excess liquid was removed and 1 mL of TRI REAGENT™ was added to each sample. Samples were mixed by hand for 15 s and allowed to stand for 2 – 15 min at room temperature. The resulting mixture was centrifuged at 12000 × g for 15 min at 4°C. The aqueous phase was transferred to a fresh microcentrifuge tube and an equal amount of 70% ethanol was added. Samples were transferred to a PureLink™ Micro-to-Midi Total RNA Purification System column (Invitrogen) and processed according to the manufacturer's protocol. All samples were washed from the column with 75 μl of RNAse free water.

### Analysis of RNA

The quality of RNA was checked on a Bioanalyzer 2100 (Agilent, Santa Clara, USA) using RNA 6000 Nano Labchip (Agilent, Santa Clara, USA) and 6000 RNA ladder as reference (Ambion, Austin, USA). The concentration and quantity of RNA were determined with ND-1000 (Nanodrop, Wilmington, USA).

### Preparation of aaRNA

Preparation of aaRNA was performed with an Amino Allyl MessageAmp™ II aRNA Amplification Kit (Ambion) according to the manufacturer's recommendations. For each hybridization, we labelled 5 μg of non-exposed cells (Cy3) and 5 μg of cells exposed to either ECT or EGT pulses (Cy5) mRNA. After removing the excess dye, the RNAs were dissolved in Nexterion Hybridization solution (Schott Nexterion, Jena, Germany).

### Microarrays

Microarrays were prepared with Human Apoptosis Subset v2.0 and Human Cancer Subset v3.0 (Operon, Ebersberg, Germany) 70 mer oligonucleotides and Nexterion 70 mer Oligo Microarraying Kit (Schott Nexterion) slides. A single array contained 2698 different genes, each gene being replicated at least 4 times on each array. Oligonucleotides were spotted using an MG1000 spotter (MicroGrid, Boston, USA), immobilised and stored according to the manufacturer's instructions (Schott Nexterion). All hybridisations were performed on HS400 in duplicate (Tecan, Salzburg, Austria) according to the manufacturer's instructions (Schott Nexterion). We used an LS200 scanner (Tecan) at 6 μm resolution for scanning the microarrays.

### Data analysis

We used Array-Pro Analyzer 4.5 (Media Cybernetics, Bethesda, USA) for feature extraction after imaging of microarrays. Acuity (Molecular devices, USA) was used for the filtration of bad signals, LOWESS fit and microarray data analysis. Features showing a signal intensity of more than 65000 were flagged as bad. Features with a signal less than 2 times the intensity of the background or coefficient of variation (CV, ratio between standard deviation of the background and the median feature intensity) greater than 0.3 were considered not significantly expressed and were filtered out. Log_2 _ratios were normalized using LOWESS fit [17] and the median of four replicates was used to calculate the average gene expression for a single sample. We filtered out genes that were not expressed in all replicate samples at least 1.5 times.

The Gene Ontology Tree Machine [18] program was used for gene enrichment analysis. All other statistical analyses were done using SPSS 16 (SPSS inc., Chicago, USA).

## Results

### Cell survival after electroporation

After electroporation of cells, cell viability was assessed by clonogenic assay. Using this method, we determined a 68.8% average survival rate for cells exposed to ECT pulses and a 31.4% average survival rate for cells exposed to EGT pulses.

### Microarrays

The difference in expression of genes involved in cancer development was obtained by comparison of malignant melanoma cells exposed to EGT or ECT pulses against the same untreated malignant melanoma cells. In our experimental design, microarrays with 2698 different genes were used as a dual colour system in which exposed and non-exposed cells' mRNA were separately labelled, mixed and hybridised together on each array. Only microarrays expressing at least 50% of genes were used for further analysis. All oligonucleotides on the same array were spotted in quadruplicate and each microarray analysis was performed in duplicate, thus obtaining eight measurements of the same oligonucleotide. The acquired data were analysed with Acuity 4.0 to select reliable signals. Only genes, 1266 for ECT pulses and 1805 for EGT pulses, present in both duplicated microarrays were considered for further processing. We next checked the variability of replicate measurements on Operon's microarray platform. The average standard deviation of the Log2 ratio (treated/untreated) of replicates exposed to ECT pulses was 0.21, and 0.17 for replicates exposed to EGT pulses. This gives a standard deviation of 1.16 fold and 1.12 fold from the median value of replicates for ECT pulses and EGT pulses, respectively. Out of 2698 different genes, 7 genes showed differential expression (Table 1), when the groups of ECT and EGT pulses were combined. This represents roughly 0.26% of all genes present on a microarray. However, looking at specifically different pulsation parameters, ECT pulses yielded 34 differentially expressed genes, which is roughly 1% of the interrogated genes, whereas EGT pulses yielded 26 differentially expressed genes, again accounting for roughly 1% of all interrogated genes. When using a cut of value of 2.0, we found only 3 deregulated genes in the treatment with ECT pulses and EGT pulses (Tables 2 and 3).

**Table 1:** Genes differentially expressed in both ECT and EGT.

Name	RefSeq	Description	Median Log2 ratio
**Down**			

*RPL31*	NM_000993	RIBOSOMAL PROTEIN L31	-1.42
*CD28*	NM_006139	T CELL SPECIFIC SURFACE GLYCOPROTEIN	-0.78
*H4FN*	NM_175054	HISTONE H4	-0.72

**Up**			

	NM_014486	NEURONAL THREAD PROTEIN	0.58
*HSPA1B*	NM_005346	HEAT SHOCK 70 KDA PROTEIN 1	0.83
*CDC25C*	NM_001790	M PHASE INDUCER PHOSPHATASE 3	0.70
*CCNF*	NM_001761	G2/MITOTIC SPECIFIC CYCLIN F	0.79

**Table 2:** Genes differentially expressed using ECT.

Name	RefSeq	Description	Median Log2 ratio
**Down**			

*RPL31*	NM_000993	RIBOSOMAL PROTEIN L31	-1.52
*RPS17*	NM_001021	40S RIBOSOMAL PROTEIN S17	-1.09
*TBCA*	NM_004607	TUBULIN SPECIFIC CHAPERONE A	-1.08
*PPIA*	NM_021130	PEPTIDYLPROLYL CISTRANS ISOMERASE A	-0.97
*S*100*B*	NM_006272	S100 PROTEIN, BETA CHAIN	-0.94
	NM_006471	MYOSIN REGULATORY LIGHT CHAIN 2	-0.93
*RPA3*	NM_002947	REPLICATION PROTEIN A 14 KDA SUBUNIT	-0.89
*NQO1*	NM_000903	NAD(P)H DEHYDROGENASE [QUINONE] 1	-0.88
*RPS6*	NM_001010	40S RIBOSOMAL PROTEIN S6	-0.81
*H4FN*	NM_175054	HISTONE H4	-0.80
*EEF1A1*	NM_001402	ELONGATION FACTOR 1 ALPHA 1	-0.77
*ITGB4*	NM_000213	INTEGRIN BETA4 PRECURSOR	-0.76
*CD28*	NM_006139	T CELL SPECIFIC SURFACE GLYCOPROTEIN CD28	-0.75
*H*3*F*3*A*	NM_002107	HISTONE H3.3	-0.74
*CASP9*	NM_001229	CASPASE 9 PRECURSOR	-0.73
*TNFRSF14*	NM_003820	TUMOR NECROSIS FACTOR RECEPTOR SUPERFAMILY MEMBER 14	-0.65
*CGB5*	NM_033142	CHORIOGONADOTROPIN BETA CHAIN PRECURSOR	-0.64
*RPH3AL*	NM_006987	RABPHILIN 3 ALIKE	-0.61
*TFDP1*	NM_007111	TRANSCRIPTION FACTOR DP-1	-0.60
*CST3*	NM_000099	CYSTATIN C PRECURSOR	-0.59

**Up**			

*RB1*	NM_000321	RETINOBLASTOMA 1	0.55
*RIN2*	NM_018993	RAS ASSOCIATION (RALGDS/AF6) DOMAIN CONTAINING PROTEIN	0.56
*DNAJB1*	NM_006145	DNAJ HOMOLOG SUBFAMILY B MEMBER 1	0.56
*MATR3*	NM_018834	MATRIN 3	0.57
*HOXA4*	NM_002141	HOMEOBOX PROTEIN HOXA4	0.65
*RBBP4*	NM_005610	CHROMATIN ASSEMBLY FACTOR 1 SUBUNIT C	0.70
*CDC25C*	NM_001790	M PHASE INDUCER PHOSPHATASE 3	0.70
	NM_014486	NEURONAL THREAD PROTEIN	0.73
*GLIPR1*	NM_006851	GLIOMA PATHOGENESIS RELATED PROTEIN	0.75
*CRABP2*	NM_001878	RETINOIC ACID BINDING PROTEIN II	0.77
*RBL2*	NM_005611	RETINOBLASTOMA LIKE PROTEIN 2	0.78
*CCNF*	NM_001761	G2/MITOTIC SPECIFIC CYCLIN F	0.79
*HSPA1B*	NM_005346	HEAT SHOCK 70 KDA PROTEIN 1	0.89
*IL6*	NM_000600	INTERLEUKIN 6 PRECURSOR (IL6)	0.98

**Table 3:** Genes differentially expressed using EGT.

Name	RefSeq	Description	Median Log2 ratio
**Down**			

*RET*	NM_020975	PROTOONCOGENE TYROSINEPROTEIN KINASE RECEPTOR	-1.364
*RPL31*	NM_000993	RIBOSOMAL PROTEIN L31	-1.218
*PC*	NM_022172	PYRUVATE CARBOXYLASE	-1.009
	NM_006590	SNRNP ASSEMBLY DEFECTIVE 1 HOMOLOG	-0.931
*IGFALS*	NM_004970	INSULIN-LIKE GROWTH FACTOR BINDING PROTEIN	-0.895
*CD28*	NM_006139	T CELL SPECIFIC SURFACE GLYCOPROTEIN	-0.845
*CYP2A7*	NM_000764	CYTOCHROME P450 2A7	-0.844
*POLR2F*	NM_021974	DNA DIRECTED RNA POLYMERASE II	-0.840
	NM_007013	WW DOMAIN CONTAINING PROTEIN 1	-0.806
	NM_004881	QUINONE OXIDOREDUCTASE HOMOLOG	-0.795
*TIMP2*	NM_003255	METALLOPROTEINASE INHIBITOR 2 PRECURSOR	-0.795
	NM_005851	DOC1 RELATED PROTEIN (DOC1R)	-0.753
*H4FN*	NM_175054	HISTONE H4	-0.648
*FGFR1*	NM_023111	BASIC FIBROBLAST GROWTH FACTOR RECEPTOR 1 PRECURSOR	-0.599
*LIG3*	NM_013975	DNA LIGASE III	-0.582
*SKP2*	NM_032637	S PHASE KINASE ASSOCIATED PROTEIN 2	-0.550
*MUC4*	NM_004532	MUCIN 4, ISOFORM D	-0.549
*PSMB7*	NM_002799	PROTEASOME SUBUNIT BETA TYPE 7 PRECURSOR	-0.544
*PTPN21*	NM_007039	PROTEIN TYROSINE PHOSPHATASE	-0.539
*SULT1C1*	NM_001056	SULFOTRANSFERASE	-0.520
*HSPB2*	NM_001541	HEATSHOCK PROTEIN, BETA2	-0.519

**Up**			

	NM_014486	NEURONAL THREAD PROTEIN	0.575
*HSPA1B*	NM_005346	HEAT SHOCK 70 KDA PROTEIN 1	0.747
*CDC25C*	NM_001790	MPHASE INDUCER PHOSPHATASE 3	0.759
*TTYH1*	NM_020659	TWEETY HOMOLOG 1	0.775
*CCNF*	NM_001761	G2/MITOTICSPECIFIC CYCLIN F	0.777

**Figure 1:**
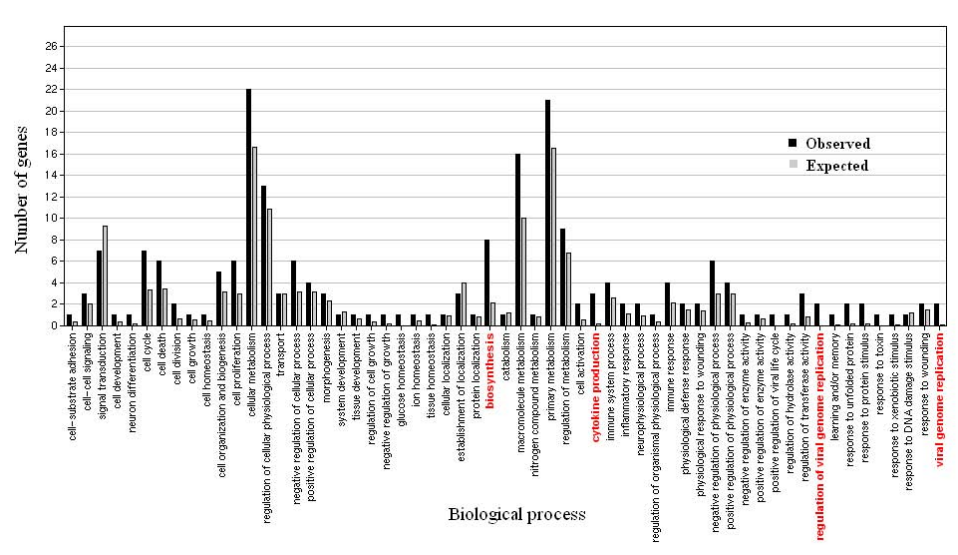
**Gene enrichment analysis for cell line exposed to ECT pulses**. Expected – number of genes expected to be differentially expressed. Observed – number of genes differentially expressed. In red are GO biological functions significantly enriched in the cell line exposed to ECT pulses.

**Figure 2:**
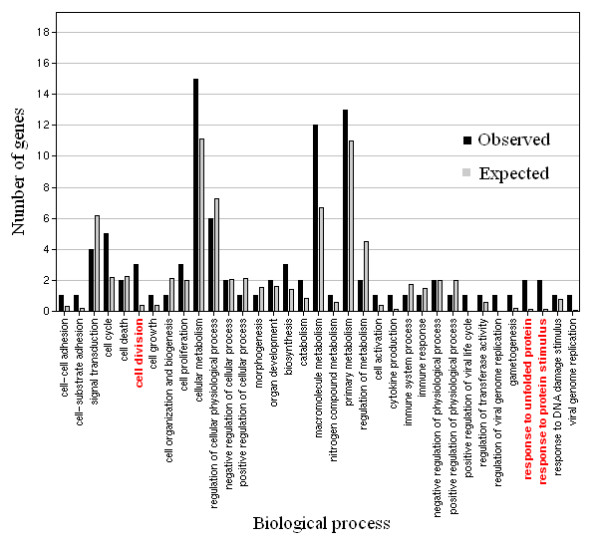
**Gene enrichment analysis for cell line exposed to EGT pulses**. Expected – number of genes expected to be differentially expressed. Observed – number of genes differentially expressed. In red are GO biological functions significantly enriched in the cell line exposed to EGT pulses.

To find the possible biological processes involved in the response to the electroporation procedure, we used the Gene Ontology Tree Machine [18] program for gene enrichment analysis. Our original dataset of genes was compared against differentially expressed genes given in Tables 2 and 3 to check whether there was any significant gene enrichment in comparison to the original gene set. Interestingly, we found significant enrichment of down-regulated genes involved in biosynthesis, regulation of viral genome replication and viral genome replication and significant enrichment of deregulated genes involved in cytokine production in melanoma cells exposed to ECT pulses (Figure 1). Deregulated genes involved in cell division, response to unfolded proteins and response to protein stimulus, were enriched in melanoma cells exposed to EGT pulses (Figure 2).

In order to enable other users comprehensively to interpret and evaluate our results, the original tables of complete microarray results are available in the supplementary data (see the GEO website at http://www.ncbi.nlm.nih.gov/projects/geo Series entry: GSE15420).

## Discussion

In this study, we analysed the expression profile of malignant melanoma cells for genes known to be involved in the development of cancer. This was done in order to assess whether electroporation could lead to an altered expression profile of cells, possibly making them more detrimental to patients. Our results show only minor differences in the expression of genes involved in cancer development. Overall, microarrays showed differential expression of only 7 genes, when using a threshold value of 1.5 fold and only 1 gene when using a threshold value of 2.0 fold (Table 1). When calculating the standard deviation of measurements across the microarray, we found it to be very low (1.16 and 1.12 fold for ECT and EGT pulses replicates) showing that the 1.5 fold threshold change is a reasonable and reliable cut-off value. The results obtained are also in agreement with studies performed so far [15,16]. However, these studies used mouse muscle cells to account for any damage to tissue or difference in expression profile made by electroporation for immunization purposes. Hojman *et al*. observed only minor histological changes and no changes in muscle performance or the gene expression profile of genes involved in cell death, inflammation or muscle regeneration [15]. Similar results were also obtained by Rubenstrunk *et al*. when they used Stress/Toxicology Atlas cDNA expression arrays. The group found only 2 genes out of 140 to be differentially expressed and concluded that electroporation does not induce expression of genes involved in stress and toxic response [16]. Therefore, despite the fact that only single cell line was used in our study, it is reasonable to expect that other cell lines would behave in similar way.

Interestingly, one of the seven differentially expressed genes is the stress response gene HSPA1B. HSPA1B is a member of the HSPA family of HSP70 proteins and is the strongest stress inducible member of the HSPA family [19]. It has been proposed that HSPA1B is a part of the molecular chaperon network that protects the proteome against environmental stress [20]. This shows that cells exposed to either of the electroporation protocols are exposed to the stress arising to some extent from a protein denaturation, and therefore over-express HSP70. This observation is also supported by the overexpression of DNAJB1 in cells treated with ECT pulses. DNAJB1, containing a conserved sequence motif (HPD) in the J domain is known to be critical for the acceleration of the ATPase activity of HSP70 [19].

Another interesting observation was downregulation of the histone protein H4 in both treatment protocols and a significant enrichment of downregulated genes involved in protein synthesis. Both results indicate a stall in DNA assembly to chromosomes and biosynthesis of proteins, which could arise from stress.

## Conclusion

Overall, our results clearly show that electroporation does not significantly changes the expression profile of major tumour suppressor genes or oncogenes of the cell cycle. Electroporation also does not change the expression of genes involved in the stability of DNA, therefore supporting the notion that electroporation is a safe method that does not promote tumorigenesis. However, in the present study, we showed that to some extent electroporation induces HSP70, resulting in the activation of the environmental stress response mechanism.

## Competing interests

The authors declare that they have no competing interests.

## Authors' contributions

VM carried out the isolation of RNA, microarray experiments, data analysis and drafted the manuscript. VT and MC performed cultivation of cells, electroporation and cell survival. VT also helped to draft the manuscript. MC, DG and GS conceived the study and participated in its design and coordination and critically revised the draft. All authors read and approved the final manuscript.

## Pre-publication history

The pre-publication history for this paper can be accessed here:

http://www.biomedcentral.com/1471-2407/9/299/prepub
